# Three-Dimensional Segmentation of Pulp Stones in Tooth-Level Volumes on Cone-Beam Computed Tomography Using nnU-Net v2: A Preliminary Study

**DOI:** 10.3390/diagnostics16193078

**Published:** 2026-09-22

**Authors:** Ezgi Katı, Gizem Eğilmez Özkılıç, Merve Yeniçeri Özata, İbrahim Şevki Bayrakdar

**Affiliations:** 1 Department of Oral and Maxillofacial Radiology, Faculty of Dentistry, Dicle University, 21280 Diyarbakır, Turkey; ezgi.kati@dicle.edu.tr (E.K.); gizem.egilmez@dicle.edu.tr (G.E.Ö.); 2 Department of Endodontics, Faculty of Dentistry, Dicle University, 21280 Diyarbakır, Turkey; 3 Department of Oral and Maxillofacial Radiology, Faculty of Dentistry, Eskisehir Osmangazi University, 26040 Eskisehir, Turkey; isbayrakdar@ogu.edu.tr

**Keywords:** artificial intelligence, cone-beam computed tomography, dental pulp stone

## Abstract

**Objectives:** This study aimed to develop and evaluate an nnU-Net version 2 (nnU-Net v2)-based model for three-dimensional segmentation of pulp stones on cone-beam computed tomography (CBCT) images, with particular emphasis on segmentation performance within preselected tooth-level volumes of interest (VOIs) known to contain pulp stones. **Methods:** This retrospective methodological study included 90 CBCT examinations in which pulp-stone presence was confirmed according to predefined eligibility criteria. Reference annotations were established through consensus among three experienced observers. Segmentation was initially evaluated on complete CBCT volumes. Subsequently, 238 pulp stone-positive tooth-level VOIs were generated and divided at the VOI level into training and held-out test datasets comprising 215 and 23 VOIs, respectively. Because the allocation was performed at the VOI level, different VOIs from the same patient could be represented in both datasets. Model performance was assessed using voxel-level overlap, classification, surface-distance, and receiver operating characteristic metrics. **Results:** The full-volume model demonstrated poor segmentation performance, with a Dice similarity coefficient (DSC) of 0.183, Jaccard index of 0.119, precision of 0.254, and recall of 0.205. Within the held-out VOI-level test set of preselected pulp stone-positive tooth VOIs, the model achieved a DSC of 0.637, Jaccard index of 0.485, precision of 0.695, and recall of 0.635. The average surface distance and average symmetric surface distance were 0.256 mm and 0.286 mm, respectively. Voxel-level accuracy was 0.998, and the area under the Receiver Operating Characteristic (ROC) curve was 0.967. However, these classification metrics represented pulp stone-versus-background voxel classification within positive tooth VOIs and did not represent tooth-level diagnostic performance. **Conclusions:** The nnU-Net v2 model demonstrated moderate segmentation performance within preselected pulp stone-positive tooth-level VOIs, whereas the poor full-volume results showed that reliable automatic localization of pulp stones in complete CBCT examinations remains unresolved. The findings provide preliminary evidence for computer-assisted pulp-stone delineation within preselected tooth volumes but do not establish an end-to-end detection approach or clinical readiness. Further optimization and external validation using both pulp stone-positive and pulp stone-negative teeth are required.

## 1. Introduction

Pulp calcifications are calcified structures that develop within the dental pulp and are most commonly detected incidentally on radiographic examinations performed for other clinically justified indications. Based on their morphological characteristics, they are classified as pulp stones (denticles) or diffuse pulp calcifications. Histologically, pulp stones are further categorized as true pulp stones, which exhibit tubular dentin surrounded by odontoblast-like cells, and false pulp stones, which result from the mineralization of degenerative cellular remnants. Pulp stones may occur freely within the pulp chamber, be attached to dentin, or become embedded within dentin. They also exhibit considerable variability in number, size, shape, and location [1,2].

The reported prevalence of pulp stones varies widely, ranging from 8% to 95%, depending on the imaging modality, study population, and diagnostic criteria used. Cone-beam computed tomography (CBCT)-based studies conducted in the Turkish population have reported that more than half of individuals present with at least one pulp stone [3,4,5]. Although pulp stones are usually asymptomatic, they may complicate endodontic treatment by hindering access cavity preparation, canal orifice identification, and root canal instrumentation, thereby prolonging treatment time and increasing the risk of procedural complications. Furthermore, growing evidence investigating their potential association with cardiovascular diseases, nephrolithiasis, and other ectopic calcifications has increased interest in pulp stones as possible indicators of systemic calcification processes rather than merely localized dental findings [6,7,8].

Periapical and bitewing radiographs have traditionally been used for the radiographic assessment of pulp stones. However, these two-dimensional imaging techniques are inherently limited by anatomical superimposition, geometric distortion, and lack of buccolingual information. Consequently, small calcifications may be difficult to distinguish from the surrounding dentin, leading to interobserver variability in diagnosis. In addition, pulp calcifications smaller than approximately 200 μm are reported to be difficult to detect reliably on conventional radiographs [3,9,10].

CBCT provides high-resolution three-dimensional imaging with submillimeter isotropic voxels, enabling detailed assessment of the location, morphology, and relationship of pulp stones to the pulp chamber and root canal system [3,4,9]. By eliminating anatomical superimposition and allowing multiplanar visualization, CBCT offers substantially greater diagnostic information than conventional two-dimensional radiography for the evaluation of pulp anatomy. Nevertheless, manual interpretation of CBCT volumes remains time-consuming, observer-dependent, and requires considerable expertise. Given the rapidly increasing number of CBCT datasets stored in digital archives, there is a growing need for automated image analysis methods [11].

In recent years, artificial intelligence (AI), particularly deep learning-based convolutional neural networks, has demonstrated remarkable success in dental imaging. Models developed using CBCT and panoramic radiographs have achieved high performance in the automatic detection and segmentation of teeth, the mandibular canal, the upper airway, the maxillary sinus, periapical lesions, and other anatomical structures [12]. In contrast, studies on pulp stones have largely focused on object detection or two-dimensional segmentation using panoramic radiographs, whereas three-dimensional automatic segmentation of pulp stones on CBCT images remains scarce [5,13,14,15]. Because pulp stones are typically small, morphologically heterogeneous, and exhibit radiodensity similar to that of the surrounding dentin, they represent one of the most challenging targets for automated segmentation in dental imaging.

Therefore, the development of three-dimensional segmentation approaches may support the standardized and reproducible delineation of pulp stones in CBCT images and facilitate the analysis of imaging datasets [16,17]. However, the feasibility of automatically localizing pulp stones within complete CBCT examinations and segmenting them within tooth-level volumes remains insufficiently investigated.

Accordingly, the present study aimed to develop and evaluate an nnU-Net version 2 (nnU-Net v2)-based deep learning model for three-dimensional segmentation of pulp stones in CBCT images. Segmentation was initially evaluated on complete CBCT volumes to investigate the feasibility of automatic localization, followed by performance assessment within preselected tooth-level volumes of interest (VOIs) known to contain pulp stones. Model performance was evaluated on a held-out VOI-level test dataset using voxel-level overlap, surface-distance, and classification metrics. The VOI-based analysis was designed to assess segmentation within preselected pulp stone-positive tooth volumes rather than to evaluate tooth-level detection or diagnostic accuracy.

## 2. Materials and Methods

### 2.1. Study Design

This retrospective, observational, methodological diagnostic performance study was conducted to develop and evaluate an nnU-Net v2-based deep learning model for the automatic segmentation of pulp stones on CBCT images.

The dataset was constructed from CBCT examinations previously acquired for various clinical indications and archived in the Department of Dentomaxillofacial Radiology, Faculty of Dentistry, Dicle University. No additional radiographic examinations were performed, and no clinical interventions were undertaken for the purposes of this study.

Teeth containing pulp stones were identified from CBCT images that met the predefined inclusion and exclusion criteria. A reference dataset generated from manually annotated segmentation masks was subsequently used to train the nnU-Net v2 model and evaluate its performance on a held-out VOI-level test dataset [18,19].

The article for this study was prepared following the Checklist for Artificial Intelligence in Medical Imaging (CLAIM) and the Standards for the Reporting of Diagnostic Accuracy Studies (STARD) Checklist. This study was approved by the Local Ethics Committee of the Faculty of Dentistry, Dicle University (approval No. 2025-67; 25 June 2025). The requirement for informed consent was waived because of the retrospective nature of this study and the use of anonymized archival CBCT images. This study was conducted in accordance with the ethical principles of the Declaration of Helsinki.

### 2.2. CBCT Image Acquisition and Dataset Preparation

All images had been previously acquired for clinically justified indications based on individual patient needs.

All CBCT examinations were acquired using an i-CAT Next Generation CBCT system (Imaging Sciences International, Hatfield, PA, USA) at 120 kV and 5 mA, with a 16 × 13 cm field of view (FOV) and native voxel sizes ranging from 0.3 to 0.4 mm. Standardized acquisition parameters were selected according to the clinical indication and individual diagnostic needs.

Before analysis, all CBCT datasets were anonymized and assigned unique study identification numbers to ensure patient confidentiality.

### 2.3. Inclusion and Exclusion Criteria

Cases were eligible for inclusion if the presence of pulp stones was confirmed on both panoramic radiographs and CBCT images, and the CBCT scans were of sufficient diagnostic quality to allow reliable evaluation of the pulp stones. In addition, the pulp chamber and/or root canal system of the corresponding tooth had to be clearly visualized on the CBCT images. The requirement for visibility on both imaging modalities was adopted as a conservative case-selection criterion to ensure that the included calcifications could be confidently identified and matched across the paired examinations before three-dimensional annotation. This criterion was not intended to establish panoramic radiography as the definitive reference standard.

CBCT examinations were excluded if image quality was inadequate for diagnostic assessment or if they contained artifacts that could interfere with image interpretation, including motion artifacts, beam-hardening artifacts, metal artifacts caused by restorations or orthodontic appliances, scanner-related artifacts, or cone-beam artifacts.

At the tooth level, teeth were excluded if they had undergone previous root canal treatment; contained extensive restorations or prosthetic restorations; exhibited loss of pulp chamber integrity due to caries or restorative procedures; showed severe coronal destruction, external or internal root resorption, or incomplete root development; or presented with periapical or periodontal pathologies that could compromise the evaluation of the pulp chamber or root canal system.

A total of 570 CBCT examinations were reviewed. After applying the inclusion and exclusion criteria, 303 examinations were excluded, and 267 CBCT examinations remained eligible. Among these, the presence of pulp stones was confirmed on both panoramic radiographs and CBCT images in 90 cases, which constituted the final study cohort. The process of patient selection and dataset construction is illustrated in Figure 1.

### 2.4. Radiographic Evaluation

Radiographic evaluation was performed in two sequential stages. Archived panoramic radiographs were initially screened because paired panoramic radiographs and CBCT examinations were available in the retrospective archive. Cases showing evidence of pulp stones on panoramic images were subsequently evaluated on axial, sagittal, and coronal CBCT images using i-CAT Vision software (version 1.9.3.14; Imaging Sciences International, Hatfield, PA, USA). Final inclusion was based on confirmation using both imaging modalities.

All radiographic evaluations were performed on a 21.3-inch color LCD medical monitor (RadiForce MX217; EIZO Corporation, Hakusan, Ishikawa, Japan). A pulp stone was defined as a hyperdense calcified structure located within the pulp chamber or root canal system that could be clearly distinguished from the surrounding dentin [3,10].

CBCT images were evaluated for the presence of pulp stones by a consensus panel comprising three experienced observers: a dentomaxillofacial radiologist with 3 years of clinical experience, an endodontist with 7 years of clinical experience, and a resident in dentomaxillofacial radiology with 2 years of clinical experience. The images were jointly reviewed, and only teeth for which consensus had been reached regarding the presence of pulp stones were included in the annotation process.

### 2.5. Annotation and Reference Segmentation

CBCT scans with confirmed pulp stones were exported in Digital Imaging and Communications in Medicine (DICOM) format and imported into the CranioCatch annotation platform (CranioCatch, Eskişehir, Türkiye; available online: https://www.craniocatch.com/en/ailab). For each tooth containing a pulp stone, a three-dimensional volume of interest (VOI) encompassing the entire tooth was automatically generated using a pretrained 3D Tooth model. Because tooth morphology and dimensions varied among cases, the VOIs were not standardized to a fixed volume but were individually generated to fully include each tooth.

Pulp stones were manually segmented at the voxel level using the polygon-based annotation tool available on the CranioCatch platform [20]. The resulting segmentation masks were jointly reviewed by the endodontist and the dentomaxillofacial radiology observers. The observers did not generate separate voxel-wise segmentation masks; any differences regarding mask boundaries were resolved through joint review, and a single consensus-based reference mask was established for each included tooth. The final consensus annotations were used as the reference standard for model training and performance evaluation.

### 2.6. Dataset Preparation and VOI Generation

The machine learning task addressed in this study was formulated as a three-dimensional semantic segmentation problem, in which each voxel of a CBCT volume was classified as either “Pulp Stone” or “Background.” Accordingly, an nnU-Net v2 model was developed to perform automatic voxel-wise segmentation of pulp stones.

Model development followed a predefined two-stage experimental design. In the preliminary stage, segmentation performance was evaluated using the original full-volume CBCT scans. In the second stage, the prespecified tooth-level VOI strategy was evaluated to determine whether restricting the input to the target anatomical region improved segmentation performance. The primary performance analyses reported in this study were based on the VOI-based model.

For the preliminary full-volume analysis, the 90 CBCT examinations were divided at the patient level into training and test datasets, comprising 81 and 9 CBCT examinations, respectively. Each patient and corresponding CBCT examination was assigned exclusively to one dataset.

For each tooth with a confirmed pulp stone, the manually generated ground-truth segmentation mask was transferred to the corresponding three-dimensional tooth-level VOI. Because multiple pulp stone-positive teeth could be present within a single CBCT examination, the tooth-level dataset comprised 238 VOIs obtained from 90 CBCT examinations. All VOIs in this dataset were derived from teeth already known to contain at least one pulp stone; no pulp stone-negative tooth VOIs were included. Therefore, the VOI-based analysis evaluated voxel-wise segmentation within preselected pulp stone-positive tooth volumes rather than tooth-level detection of pulp stone-positive and pulp stone-negative teeth.

For the tooth-level VOI analysis, the 238 cropped VOIs were divided at the VOI level using an approximately 90:10 ratio, resulting in 215 VOIs for model training and 23 VOIs for testing. The split was based on individual tooth-level VOIs rather than patients. Therefore, when a patient contributed more than one pulp stone-positive tooth, different VOIs from the same patient could be allocated to both the training and test datasets. The 23 test VOIs themselves were excluded from model training and were used only for final performance evaluation; however, patient-level independence between the training and test datasets was not ensured.

All CBCT volumes were converted from the DICOM format to the Neuroimaging Informatics Technology Initiative (NIfTI) format and organized according to the nnU-Net v2 dataset structure as Dataset312_Pulp_Stone_Tooth. Voxels corresponding to pulp stones were assigned to the “Pulp Stone” class, whereas all remaining voxels were labeled as “Background.”

### 2.7. Image Preprocessing and nnU-Net v2 Training

Automatic segmentation was performed using the three-dimensional full-resolution (3D full-resolution) configuration of nnU-Net v2 (version 2.8.1). nnU-Net v2 is a self-configuring U-Net-based segmentation framework that automatically determines preprocessing strategies, network architecture, patch size, and batch size by analyzing the characteristics of the input dataset, including image dimensions, voxel spacing, and class distribution.

Image preprocessing was performed using the default DefaultPreprocessor implemented in nnU-Net v2. All tooth-level VOIs were resampled to an isotropic voxel spacing of 0.30 × 0.30 × 0.30 mm. After resampling, the median VOI size was 74 × 49 × 50 voxels. Image intensities were normalized using the CT normalization strategy automatically selected by nnU-Net v2. Third-order cubic interpolation was applied for image resampling, whereas nearest-neighbor interpolation was used for the segmentation masks to preserve class labels during the resampling process. Based on the characteristics of the dataset and the available GPU memory, nnU-Net v2 automatically determined a patch size of 80 × 56 × 56 voxels and a batch size of 8.

Model training and inference were performed on a high-performance computing workstation equipped with an Intel^®^ Xeon^®^ Gold 5218 CPU (2.30 GHz; Intel Corporation, Santa Clara, CA, USA), an NVIDIA Tesla V100 PCIe GPU (16 GB VRAM; NVIDIA Corporation, Santa Clara, CA, USA), and 220 GB of system RAM. Image preprocessing, model training, automatic segmentation, and performance evaluation were conducted within the same software and hardware environment.

The nnU-Net v2 model was trained for 1000 epochs using the default optimization strategy, loss function, and online data augmentation pipeline. Training was performed using the fold = all configuration, whereby all 215 training VOIs were used for model fitting. No separate internal validation subset was created, and no validation-based model selection was performed. After completion of training, the final trained model was evaluated on the held-out test dataset comprising 23 tooth-level VOIs that had not been used for model fitting. Because the dataset was partitioned at the VOI level, patient-level independence between the training and test datasets was not ensured.

### 2.8. Model Performance Evaluation

The segmentation performance of the trained nnU-Net v2 model was evaluated using a held-out VOI-level test dataset comprising VOIs that had not been used for model fitting [18]. Model-generated segmentation masks were compared voxel-wise with the consensus-based reference segmentation masks. Sensitivity, specificity, accuracy, and ROC–AUC were calculated at the voxel level within preselected tooth-level VOIs known to contain pulp stones. Sensitivity represented the proportion of reference pulp-stone voxels correctly classified as pulp stone, whereas specificity represented the proportion of background voxels within these positive VOIs correctly classified as background. Because the VOI dataset did not include pulp stone-negative teeth, these metrics did not represent tooth-level diagnostic sensitivity, specificity, accuracy, or discrimination.

Performance evaluation was based on the number of true-positive (TP), false-positive (FP), false-negative (FN), and true-negative (TN) voxels. TP represented voxels classified as pulp stones in both the reference segmentation and the model prediction. FP represented background voxels incorrectly classified as pulp stones by the model, whereas FN represented reference pulp-stone voxels incorrectly classified as background. TN represented background voxels correctly classified as background in both segmentations.

Segmentation performance was quantified using the Dice similarity coefficient (DSC), Jaccard index, accuracy, precision, recall (sensitivity), specificity, false discovery rate (FDR), and false-negative rate (FNR). The DSC and Jaccard index were used to evaluate spatial overlap between the predicted and reference segmentations, whereas accuracy, precision, recall, specificity, FDR, and FNR were used to assess voxel-level classification performance [21].

Receiver operating characteristic (ROC) analysis was additionally performed to assess voxel-level discrimination between pulp-stone and background voxels within the preselected pulp stone-positive tooth VOIs. The area under the ROC curve (AUC) was calculated as a summary measure of this voxel-level discrimination [22]. The resulting AUC did not represent tooth-level discrimination between pulp stone-positive and pulp stone-negative teeth.

To assess geometric agreement between the predicted and reference segmentations, boundary-based distance metrics were calculated, including the average surface distance (ASD), average symmetric surface distance (ASSD), Hausdorff distance (HD), and 95th percentile Hausdorff distance (HD95). ASD and ASSD quantified the average boundary discrepancy between the predicted and reference segmentations, whereas HD measured the maximum boundary deviation and HD95 provided a more robust estimate by reducing the influence of extreme outlier distances [23]. The 95% confidence interval (CI) for the mean VOI-level Dice similarity coefficient was calculated using patient-level cluster bootstrap resampling with 10,000 repetitions. Resampling was performed at the patient level to account for the inclusion of more than one test VOI from some patients, and the confidence interval was derived from the 2.5th and 97.5th percentiles of the bootstrap distribution.

## 3. Results

### 3.1. Preliminary Analysis on Full-Volume CBCT Segmentation

As the first step of the predefined two-stage modeling strategy, segmentation performance was preliminarily evaluated using the original full-volume CBCT scans from 90 cases with confirmed pulp stones. This analysis was performed to assess the feasibility of automatic segmentation on entire CBCT volumes before implementing the planned tooth-based VOI approach. The findings obtained from this preliminary analysis supported the adoption of the VOI-based strategy as the primary modeling approach for the subsequent experiments.

The preliminary full-volume analysis yielded a Dice similarity coefficient of 0.183 and a Jaccard index of 0.119, indicating poor spatial overlap between the predicted and reference segmentations. The model achieved a precision of 0.254 and a recall of 0.205, demonstrating that reliable localization and segmentation of pulp stones could not be achieved directly within complete CBCT volumes. Supplementary voxel-level ROC analysis yielded an AUC of 0.60, further indicating limited discrimination between pulp-stone and background voxels in the full-volume setting. The overall voxel-level accuracy was 0.99998; however, this value was strongly influenced by the predominance of background voxels and should not be interpreted as evidence of satisfactory segmentation performance. Boundary-based evaluation yielded an ASD of 14.89 mm, an ASSD of 14.39 mm, an HD of 41.20 mm, and an HD95 of 39.94 mm. These findings demonstrate the limitations of the full-volume approach and support the planned evaluation of segmentation performance within preselected tooth-level VOIs. Detailed performance metrics and additional results of the full-volume model are provided in Supplementary Table S1 and Supplementary Figures S1–S3.

### 3.2. Voxel-Wise Classification Performance of the VOI-Based Model

Across the held-out VOI-level test dataset, the mean number of TP, TN, FP, and FN voxels was 266.17, 131,881.78, 135.22, and 154.52, respectively. At the voxel level, the model achieved a recall (sensitivity) of 0.635, specificity of 0.999, false-positive rate of 0.001, and false-negative rate of 0.365 within the preselected pulp stone-positive tooth VOIs. Thus, most background voxels were correctly classified, whereas 36.5% of the reference pulp-stone voxels were classified as background. These metrics reflect voxel-level pulp stone-versus-background classification within positive tooth VOIs and do not represent tooth-level diagnostic performance. The voxel-wise classification performance of the VOI-based model is presented in Table 1.

### 3.3. Segmentation Performance of the VOI-Based Model

The segmentation performance of the nnU-Net v2 model was evaluated using a held-out VOI-level test set comprising 23 tooth-level VOIs obtained from 19 individual patients. None of these 23 VOIs were used for model training. However, because dataset partitioning was performed at the VOI level, other tooth-level VOIs from some of the same patients could have been included in the training dataset. Of the 23 test VOIs, 15 were obtained from maxillary teeth and 8 from mandibular teeth, while 1 represented an anterior tooth and 22 represented posterior teeth. Because of this markedly unbalanced distribution, particularly the inclusion of only one anterior VOI, subgroup performance comparisons according to dental arch or tooth location were not performed. The model achieved a mean Dice similarity coefficient of 0.637 (95% CI: 0.560–0.693), a Jaccard index of 0.485, a precision of 0.695, a recall of 0.635, and an overall voxel-wise accuracy of 0.998. The reported accuracy reflects voxel-level pulp stone-versus-background classification within preselected pulp stone-positive tooth VOIs and should not be interpreted as tooth-level diagnostic accuracy.

Boundary-based evaluation yielded an ASD of 0.256 mm, an ASSD of 0.286 mm, an HD of 1.085 mm, and a 95th percentile Hausdorff distance (HD95) of 0.632 mm. The quantitative segmentation performance metrics are presented in Table 2.

### 3.4. Distribution of Dice Similarity Coefficients Across the Test Cases

The distribution of Dice similarity coefficients for the 23 tooth-level VOIs in the held-out test set is shown in Figure 2. The case-wise Dice scores ranged from 0.000 to 0.838, with a mean Dice similarity coefficient of 0.637. The highest Dice score achieved by the model was 0.838. In one test case, no overlap was observed between the reference and predicted segmentations, resulting in a Dice similarity coefficient of 0.000.

### 3.5. Model Training Process

The training process of the nnU-Net v2 model over 1000 epochs is illustrated in Figure 3, including changes in training loss, pseudo Dice, epoch duration, and learning rate. Throughout training, the loss values showed a progressive decrease, whereas the pseudo Dice values exhibited a corresponding increasing trend. The epoch duration remained relatively stable across the training process, while the learning rate gradually decreased according to the predefined learning schedule.

### 3.6. Receiver Operating Characteristic (ROC) Analysis

Voxel-level discrimination between pulp-stone and background voxels within the preselected pulp stone-positive tooth VOIs was evaluated using ROC analysis. The model achieved an area under the ROC curve (AUC) of 0.967. This value represents voxel-level discrimination within positive tooth VOIs and should not be interpreted as tooth-level diagnostic discrimination between pulp stone-positive and pulp stone-negative teeth. The ROC curve is presented in Figure 4.

### 3.7. Representative Segmentation Examples

Three representative cases from the held-out VOI-level test set are presented in Figure 5 to illustrate variability in model performance. Cases with high (Dice = 0.838), moderate (Dice = 0.649), and low (Dice = 0.462) segmentation performance are shown. For each case, the original cropped CBCT image, manually generated ground-truth segmentation, and corresponding model prediction are presented side by side.

## 4. Discussion

In the present study, the full-volume nnU-Net v2 model demonstrated poor segmentation performance, with a Dice similarity coefficient of 0.183, precision of 0.254, recall of 0.205, and a supplementary voxel-level AUC of 0.60. These findings indicate that reliable automatic localization and segmentation of pulp stones within complete CBCT examinations were not achieved. This result is particularly relevant because the full-volume approach more closely represents the intended clinical scenario, in which the location of a pulp stone-positive tooth is not known in advance. Restricting the analysis to preselected tooth-level VOIs improved model performance; however, the resulting Dice similarity coefficient of 0.637, precision of 0.695, and recall of 0.635 indicated moderate segmentation performance. Nevertheless, the observed false-positive and false-negative predictions demonstrate that challenges remain in the segmentation of small and morphologically heterogeneous pulp stones. Although the VOI-based model achieved high overall voxel-level accuracy, this value was strongly influenced by the predominance of background voxels, as pulp stones occupied only a very small proportion of each tooth-level VOI [24,25]. Therefore, model performance should not be interpreted solely on the basis of accuracy; overlap, error-based, and surface-distance metrics should be considered together.

The poor performance of the full-volume model most likely reflects the extreme imbalance between the small pulp-stone foreground and the substantially larger CBCT background. Because pulp-stone voxels constitute only a minute proportion of a complete CBCT volume, small calcifications may have been insufficiently represented during patch-based training and partially lost or blurred during image resampling. Moreover, the 0.3–0.4 mm voxel size meant that some pulp stones were represented by only a few voxels. Partial-volume effects, CBCT noise, poorly defined lesion boundaries, and radiodensity similarities between pulp stones and adjacent dentin or other hyperdense structures may have further contributed to false-negative and false-positive predictions. These factors provide plausible explanations for the low recall and spatial-overlap metrics, although their individual contributions could not be quantified in the present study.

The marked difference between specificity and sensitivity reflects the severe voxel-level class imbalance and the model’s conservative foreground prediction. The predominance of background voxels resulted in relatively few false-positive predictions and very high specificity, whereas the small size and indistinct boundaries of pulp stones increased false-negative predictions and reduced sensitivity. Importantly, specificity and accuracy represent voxel-level pulp stone-versus-background classification within positive VOIs, not the identification of pulp stone-negative teeth. Similarly, the ROC AUC reflects voxel-level discrimination within positive VOIs and cannot establish tooth-level discrimination between pulp stone-positive and pulp stone-negative teeth.

Although restricting the input to tooth-level VOIs reduced irrelevant background and improved overlap metrics, it required prior identification and localization of the tooth containing the pulp stone. Therefore, the VOI-based model does not constitute an end-to-end approach capable of detecting pulp stones directly from complete CBCT examinations. Accordingly, the findings represent preliminary evidence of computer-assisted pulp-stone delineation within preselected tooth volumes rather than automatic detection in complete CBCT examinations. These results should also be interpreted cautiously because VOI-level partitioning did not ensure patient-level independence between the training and test datasets.

The literature on the artificial intelligence-assisted evaluation of pulp stones remains limited and methodologically heterogeneous. Previous studies have mainly investigated classification, object detection, or two-dimensional segmentation on bitewing or panoramic radiographs [26,27,28]. Although the few CBCT-based studies have demonstrated the feasibility of three-dimensional detection or segmentation, reduced performance has been reported for small and diffusely distributed calcifications [29,30]. The present study instead evaluated voxel-wise pulp-stone segmentation within preselected tooth-level VOIs using nnU-Net v2.

Pulp stones vary considerably in size, number, morphology, and location within the pulp chamber. Their reported prevalence also differs according to the study population, tooth group, and imaging modality. Zhang et al. reported an age-related increase and a higher prevalence in maxillary teeth and molars [24], whereas Kaabi et al. found no significant association with age but identified first molars as the most frequently affected teeth [31]. Associations with caries and restorations have likewise been reported inconsistently across populations [31]. This anatomical and radiological heterogeneity complicates automatic segmentation, particularly for small calcifications, irregular lesions, and those located in different regions of the pulp space [1,13].

Imaging modality plays an important role in pulp-stone detection. Hsieh et al. detected pulp stones in 83.3% of individuals and 31.3% of examined teeth using CBCT [10], while Taşsöker et al. identified more pulp stones with CBCT than with digital panoramic radiography in the same individuals [3]. In the present retrospective study, panoramic radiographs were used only for initial screening because paired panoramic and CBCT examinations were available in the institutional archive; multiplanar CBCT images were used for three-dimensional assessment and annotation. Thus, panoramic radiography was not considered the preferred modality for definitive assessment. CBCT enables multiplanar visualization and voxel-wise segmentation, although its performance is affected by voxel size, image quality, and artifacts. Pulp-stone detection alone should not constitute an indication for CBCT; instead, AI-assisted analysis should be applied secondarily to scans acquired for other clinically justified indications [10,32]. When CBCT is indicated for endodontic assessment, a limited FOV appropriate to the region under investigation is generally preferred to restrict the irradiated volume and may permit smaller voxel sizes. Validation using limited-FOV endodontic CBCT examinations is therefore required before this approach can be considered for endodontic applications [9,33].

The Dice similarity coefficient of 0.637 indicates moderate volumetric overlap between the reference and predicted segmentations. Because pulp stones are small and often have irregular boundaries and variable radiodensity, minor boundary discrepancies can substantially reduce the Dice score. In contrast, the low ASD (0.256 mm) and ASSD (0.286 mm) indicate that the predicted boundaries were generally close to the reference contours. Thus, the model achieved partial delineation within preselected tooth VOIs, but incomplete overlap indicates that further methodological improvement is required.

One case in the held-out VOI-level test set had a Dice similarity coefficient of 0.000, indicating no overlap between the predicted and reference segmentations. Similarly, Orhan et al. reported one incorrectly identified tooth and 11 undetected lesions among 153 periapical lesions. These findings show that case-level segmentation or detection failures may occur even when overall model performance appears favorable [34].

The false-negative rate (FNR = 0.3653) indicates that approximately one-third of the reference pulp-stone voxels were missed, potentially because of incomplete segmentation of small, low-contrast calcifications or those located within narrow root canals. The false discovery rate (FDR = 0.3052) further indicates that some predicted pulp-stone voxels were false positives, reflecting over-segmentation.

Despite the exclusion of root canal-treated teeth and teeth with extensive restorations, sclerotic dentin and other hyperdense structures at the dentin–pulp interface may still have contributed to segmentation errors. Therefore, performance was influenced not only by the network architecture but also by target characteristics, image resolution, and reference-annotation quality.

Methodological differences limit direct comparison with previous AI studies. Altındağ et al. reported an F1 score (the harmonic mean of precision and recall) of 0.7892 for pulp-stone detection on panoramic radiographs using YOLOv5 [26], while Gürhan et al. achieved an F1 score of 0.966 with a YOLOv8- and ResNeXt-based approach [14]. A panoramic segmentation study using U^2^-Net reported a Dice similarity coefficient of 0.759 [28]. Although these values exceed the Dice score in the present study, two-dimensional object detection and segmentation differ fundamentally from three-dimensional voxel-wise segmentation in CBCT volumes.

Segmentation performance is strongly influenced by the size, continuity, and morphological complexity of the target structure [35,36]. For example, Dice coefficients above 0.90 have been reported for larger and more continuous structures: Sin et al. achieved a Dice coefficient of 0.919 and an accuracy of 0.961 for pharyngeal airway segmentation [37]. Lower values have been reported for smaller targets, including Dice coefficients of 0.7627 and 0.73 for mandibular canal segmentation [38,39], 0.86–0.88 for root canal systems [30], and approximately 0.39 for small soft-tissue calcifications [40]. Similar variation in segmentation performance has also been reported for other anatomical structures on CBCT images [41]. These differences may reflect variation in target size, morphology, anatomical continuity, image resolution, model architecture, and reference-annotation quality; therefore, performance across different anatomical targets should not be directly compared. The present findings provide preliminary evidence of the feasibility of three-dimensional pulp-stone segmentation within preselected tooth-level VOIs while highlighting the need for improved performance in small, low-contrast calcifications.

Potential associations between pulp stones and systemic diseases have also been investigated. Gabardo et al. reported a possible relationship with nephrolithiasis [8], while Alğan et al. evaluated associations with cardiovascular diseases, diabetes mellitus, and other systemic conditions [42]. However, the heterogeneity of current evidence prevents pulp stones from being considered a reliable radiographic marker of systemic disease [42,43]. In the future, sufficiently validated AI-assisted methods may facilitate large-scale imaging analyses of these associations and potential opportunistic-screening applications [44,45].

The main strengths of this study include voxel-wise manual segmentation on three-dimensional CBCT images, consensus-based reference annotations from three observers, use of the nnU-Net v2 framework, and comprehensive evaluation using complementary overlap, surface-distance, classification, and error-based metrics rather than accuracy alone.

The present study has several limitations. First, all data were obtained from a single institution using one CBCT scanner, and no external validation was performed. The held-out VOI-level test set contained only 23 tooth-level VOIs obtained from 19 patients, limiting the precision and generalizability of the performance estimates. Furthermore, because dataset partitioning was performed at the tooth-VOI level rather than the patient level, different VOIs from the same patient could be represented in both the training and test datasets. Although the test VOIs themselves were withheld from model training, this potential patient-level overlap may have resulted in optimistic performance estimates and may preclude conclusions regarding independent patient-level generalization.

Second, panoramic screening may have excluded small or subtle pulp stones visible only on CBCT. Anterior teeth outside the focal trough because of patient positioning or skeletal relationships may also appear blurred, while requiring visibility on both modalities may have preferentially selected larger or more radiopaque calcifications and introduced selection bias. Third, because reference annotations were established by joint consensus rather than separate observer-specific segmentations, quantitative interobserver agreement could not be assessed. Fourth, all examinations used a relatively large FOV of 16 × 13 cm with native voxel sizes of 0.3–0.4 mm. These parameters may have limited the representation of small pulp stones through partial-volume effects. Cropping tooth-level VOIs reduced irrelevant background but did not improve the intrinsic spatial resolution; therefore, the findings may not generalize to limited-FOV endodontic protocols with smaller voxels. Fifth, the poor full-volume performance shows that the model could not reliably localize and segment pulp stones within complete CBCT examinations. Although preselected tooth-level VOIs improved performance, this approach required prior identification of the affected tooth and achieved only moderate spatial overlap. Further optimization of the training strategy and model architecture was not evaluated. The findings should therefore be considered preliminary and not interpreted as evidence of readiness for independent clinical use.

The test set was anatomically unbalanced, comprising 15 maxillary and 8 mandibular VOIs, with only 1 anterior VOI compared with 22 posterior VOIs; therefore, reliable subgroup comparisons by tooth location or dental arch could not be performed. Performance was also not evaluated according to pulp-stone size, morphology, or anatomical location. Furthermore, because the VOI dataset contained only pulp stone-positive teeth, specificity, accuracy, and ROC–AUC represented voxel-level pulp stone-versus-background classification within positive VOIs rather than tooth-level diagnostic performance. The model’s ability to distinguish positive from negative teeth and its clinical diagnostic accuracy therefore remain unknown. Lesion-level metrics, direct comparison with expert performance, and prospective clinical validation were also unavailable.

Future studies should evaluate two-stage frameworks that first localize teeth and/or pulp chambers and then detect and segment pulp stones within the resulting VOIs. Including both pulp stone-positive and pulp stone-negative teeth would permit assessment of tooth-level performance and false-positive predictions, while greater representation of small calcifications and small-object-sensitive sampling and loss functions may improve segmentation. Full-volume and tooth-level VOI approaches should also be compared using large- and limited-FOV CBCT examinations with different native voxel sizes to determine whether limited-FOV imaging improves small-calcification segmentation and reduces dependence on prior tooth localization. Larger multicenter datasets from different scanners and imaging protocols should be evaluated according to pulp-stone size, morphology, tooth group, dental arch, and anatomical location using lesion- and tooth-level metrics. All methodological improvements require evaluation on independent external test datasets before clinical application.

Studies comparing two- and three-dimensional approaches on the same dataset would permit a more objective assessment of their respective advantages and limitations. Prospective studies should also compare performance and interpretation time among experts, AI, and combined expert–AI assessment to determine the potential clinical utility of the proposed approach.

## 5. Conclusions

The nnU-Net v2 model demonstrated moderate segmentation performance within preselected pulp stone-positive tooth-level VOIs. However, the poor full-volume results indicate that reliable automatic localization of pulp stones directly within complete CBCT examinations remains unresolved. Because the VOI-based analysis required prior identification of a pulp stone-positive tooth and did not include pulp stone-negative teeth, the findings should not be interpreted as evidence of automatic tooth-level detection or tooth-level diagnostic accuracy. The proposed approach should instead be regarded as preliminary evidence for computer-assisted pulp-stone delineation within preselected tooth volumes. Further model optimization and external validation using both positive and negative teeth are required before clinical application.

## Figures and Tables

**Figure 1:**
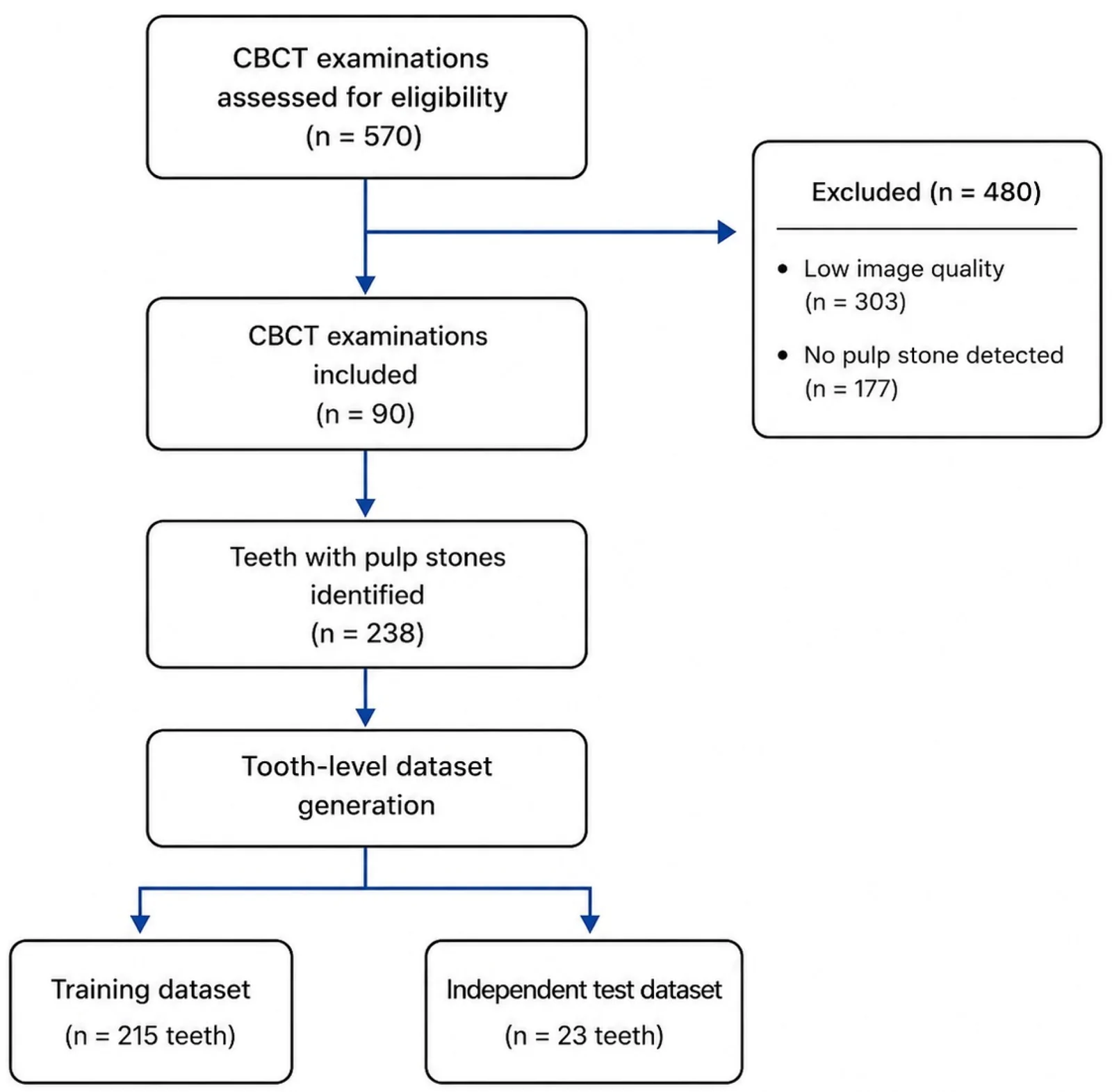
Flow diagram of CBCT examination selection and tooth-level VOI dataset generation (CBCT, cone-beam computed tomography; VOI, volume of interest). Dataset partitioning for the VOI-based analysis was performed at the tooth-VOI level rather than the patient level; consequently, different VOIs from the same patient could be allocated to both the training and held-out test datasets.

**Figure 2:**
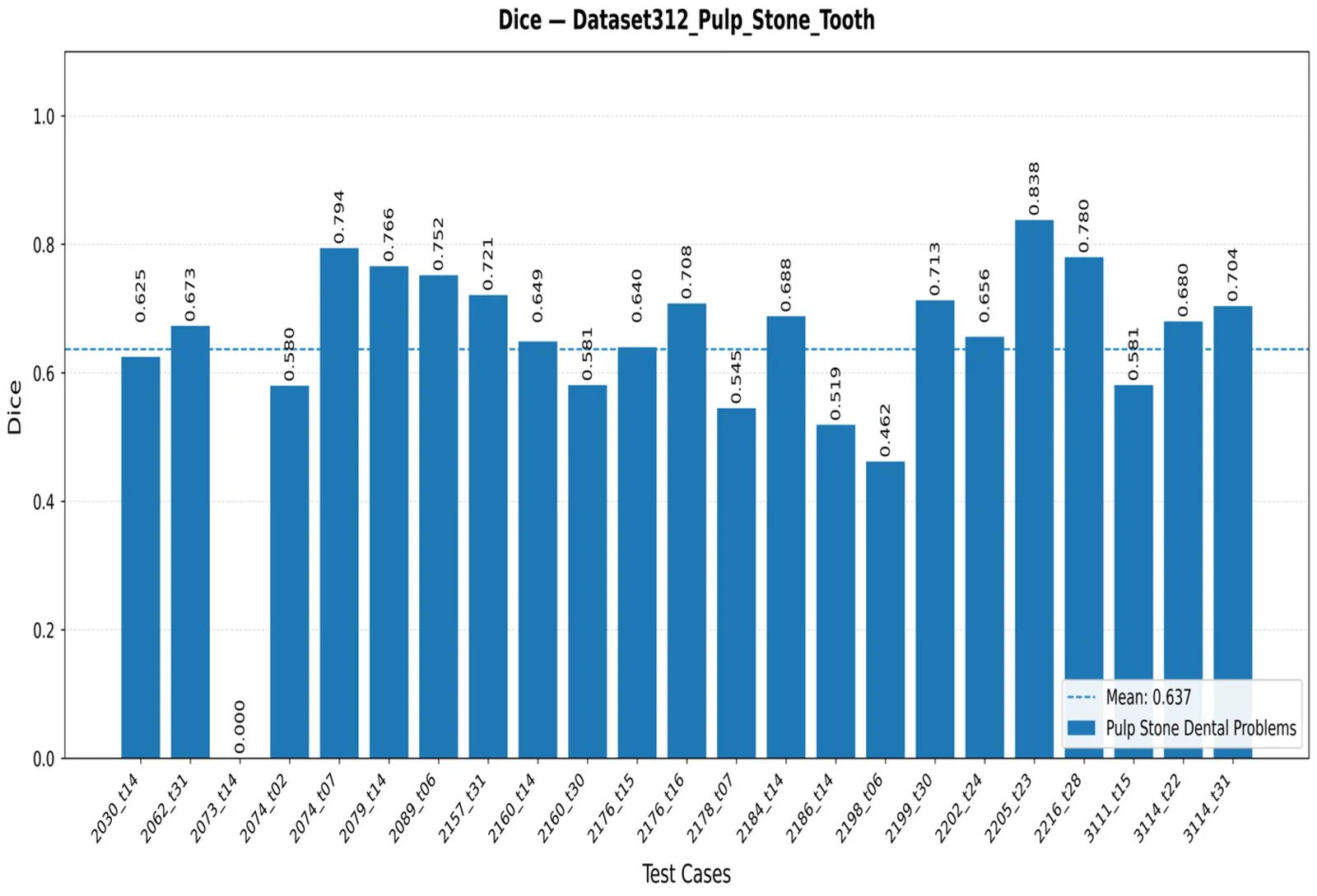
Distribution of Dice similarity coefficients across the 23 tooth-level VOIs in the held-out test set.

**Figure 3:**
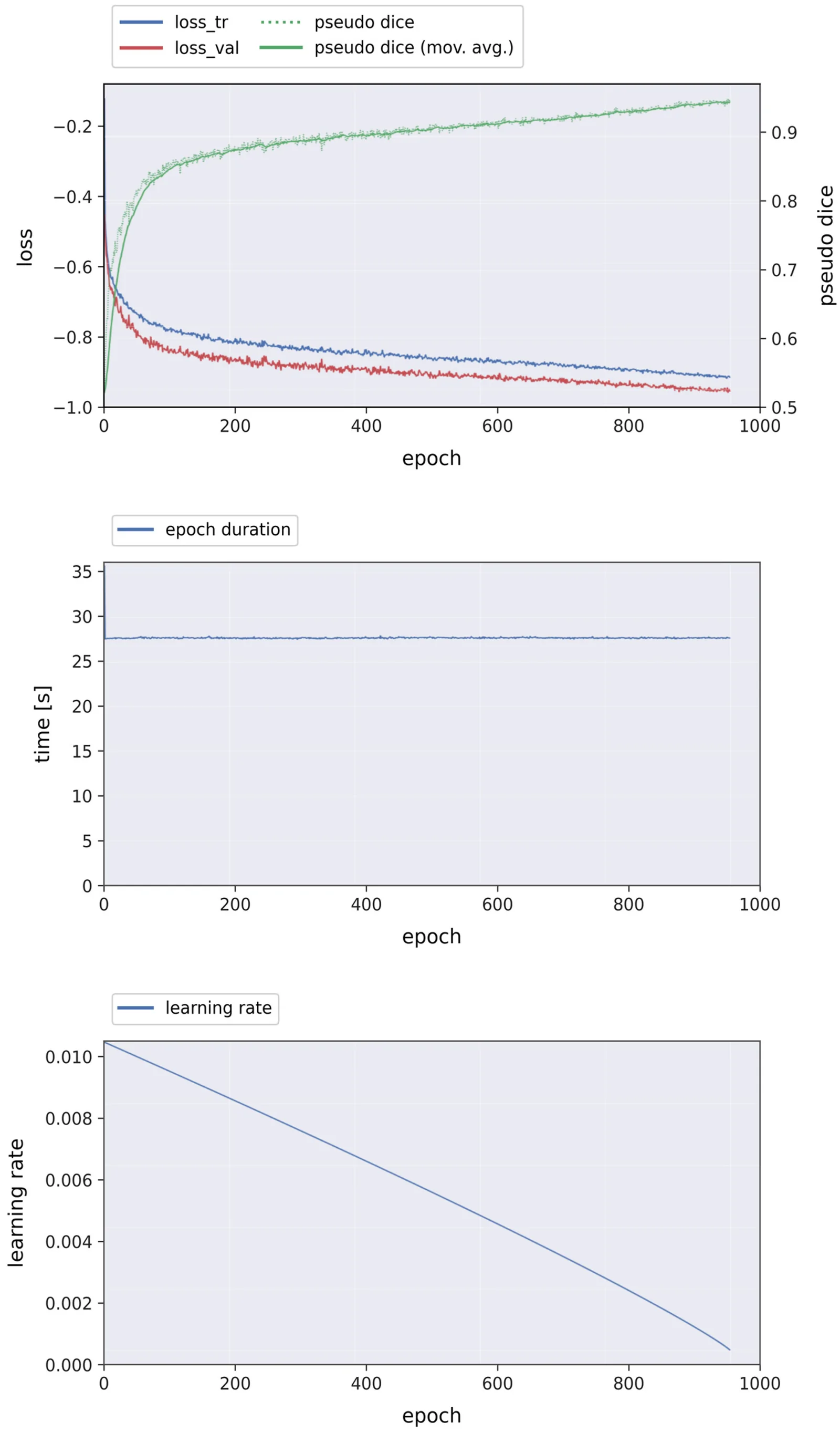
Training curves of the nnU-Net v2 model over 1000 epochs, showing the changes in training loss, pseudo Dice, epoch duration, and learning rate.

**Figure 4:**
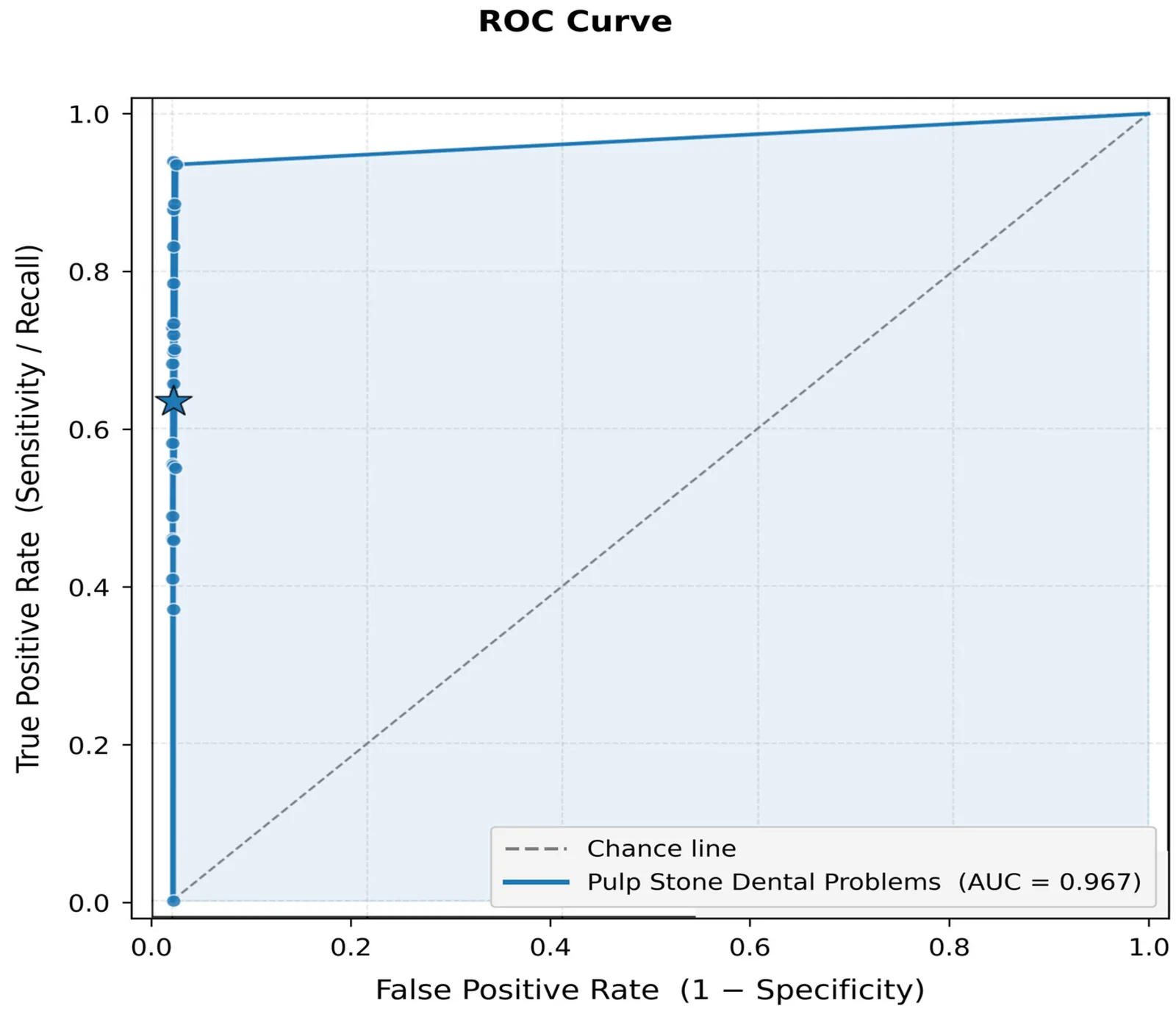
Voxel-level receiver operating characteristic (ROC) curve of the volume-of-interest (VOI)-based nnU-Net v2 model on the held-out VOI-level test set (area under the curve (AUC) = 0.967). The star indicates the operating point corresponding to a sensitivity of 0.635 and specificity of 0.999 within preselected pulp stone-positive tooth VOIs. These metrics represent pulp stone-versus-background voxel classification and not tooth-level diagnostic discrimination.

**Figure 5:**
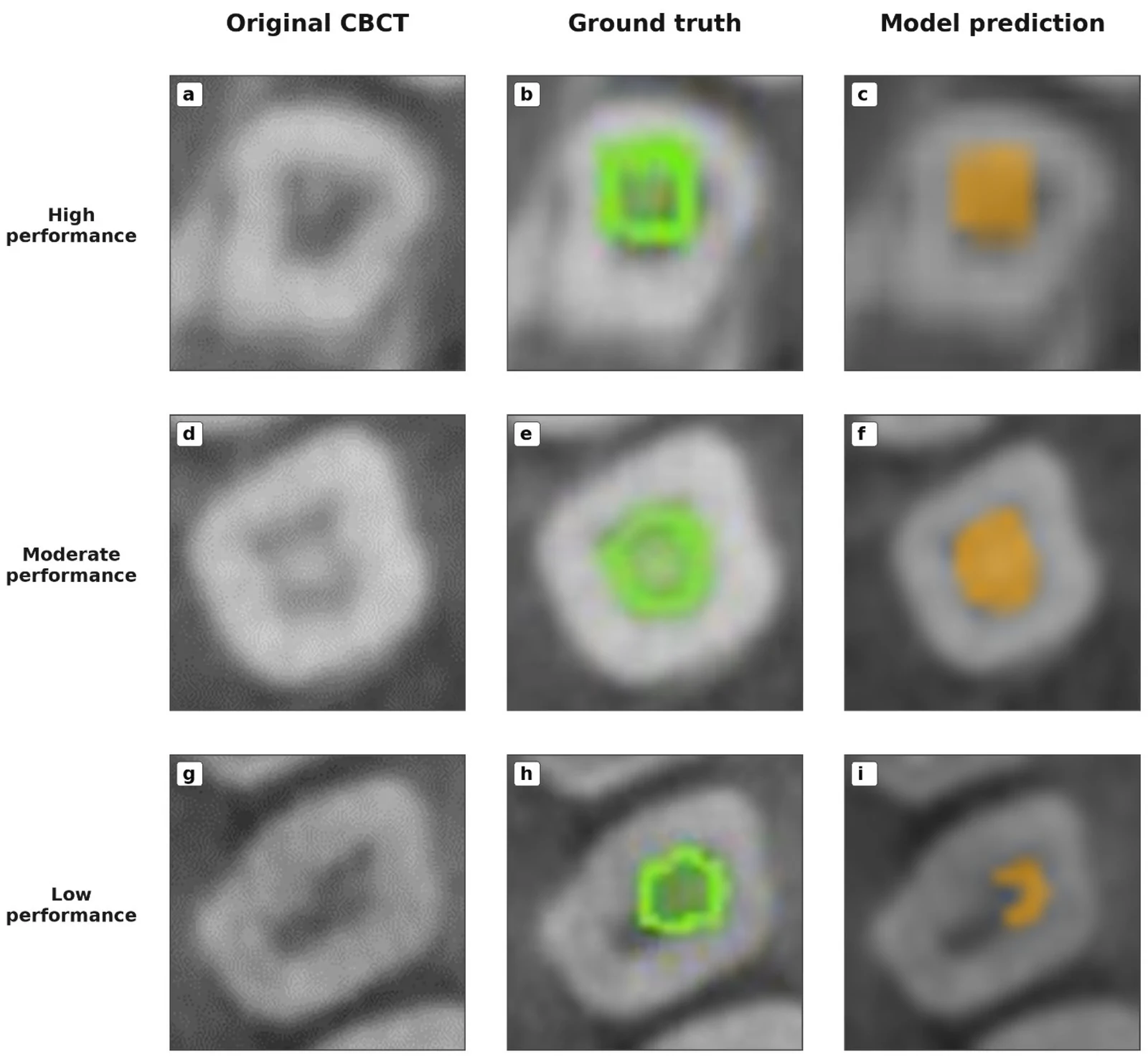
Representative segmentation results from tooth-level volumes of interest (VOIs) in the held-out test set, illustrating cases with high (**a**–**c**), moderate (**d**–**f**), and low (**g**–**i**) segmentation performance. For each case, the original cropped cone-beam computed tomography (CBCT) image, manual ground-truth segmentation, and corresponding model prediction are shown. Green indicates the manual ground-truth segmentation, and orange indicates the model prediction.

**Table 1:** Voxel-wise classification performance of the nnU-Net v2 model.

Variable	Value
True positive (TP)	266.17
True negative (TN)	131,881.78
False positive (FP)	135.22
False negative (FN)	154.52
True-positive rate/sensitivity	0.635
True-negative rate/specificity	0.999
False-positive rate	0.001
False-negative rate	0.365

All values were calculated at the voxel level within preselected pulp stone-positive tooth VOIs. TN and specificity refer to correctly classified background voxels within positive VOIs and do not represent pulp stone-negative teeth or tooth-level diagnostic specificity. (FP, false positive; FN, false negative; TN, true negative; TP, true positive; VOI, volume of interest).

**Table 2:** Segmentation performance metrics of the nnU-Net v2 Model on the held-out VOI-level test set.

Performance Metric	Value
Dice similarity coefficient	0.637
Jaccard index	0.485
Precision	0.695
Sensitivity	0.635
Average surface distance	0.256
Average symmetric surface distance	0.286
Hausdorff distance	1.085
95th percentile Hausdorff distance (HD95)	0.632
Voxel-level accuracy	0.998

Accuracy, precision, and sensitivity were calculated at the voxel level within preselected pulp stone-positive tooth VOIs. These values do not represent tooth-level diagnostic performance. (HD95, 95th percentile Hausdorff distance; VOI, volume of interest).

## Data Availability

The data presented in this study are available from the corresponding author upon reasonable request, subject to institutional restrictions.

## Supplementary Materials

The following supporting information can be downloaded at: https://www.mdpi.com/article/10.3390/diagnostics16193078/s1, Table S1: Overall segmentation performance of the full-volume CBCT model on the test set; Figure S1: Receiver operating characteristic curve of the full-volume CBCT segmentation model; Figure S2: Training dynamics of the full-volume CBCT nnU-Net v2 model; Figure S3: Case-wise Dice Similarity Coefficient values of the full-volume CBCT model.

## Abbreviations

The following abbreviations are used in this manuscript:

CBCT	Cone-beam computed tomography
DICOM	Digital Imaging and Communications in Medicine
VOI	Volume of interest
NIfTI	Neuroimaging Informatics Technology Initiative
TP	True positive
FP	False positive
FN	False negative
TN	True negative
DSC	Dice similarity coefficient
FDR	False discovery rate
FNR	False-negative rate
ROC	Receiver operating characteristic
AUC	Area under the ROC curve
ASD	Average surface distance
ASSD	Average symmetric surface distance
HD	Hausdorff distance
HD95	95th percentile Hausdorff distance
