# Supplementary material for: Three-Dimensional Segmentation of Pulp Stones in Tooth-Level Volumes on Cone-Beam Computed Tomography Using nnU-Net v2: A Preliminary Study
Source: Diagnostics (Basel). 2026 Sep 22;16(19):3078. doi: 10.3390/diagnostics16193078 (PMC13663998; doi:10.3390/diagnostics16193078)
Supplement: Supplementary file 1 [file diagnostics-16-03078-s001.zip › diagnostics-4494887-supplementary.pdf]

Supplementary Table S1. Overall segmentation performance of the full-volume CBCT model on the test set

**Performance Metric**	**Mean Value**
Dice Similarity Coefficient (DSC)	0.1829
Jaccard Index (JI)	0.1187
Accuracy	0.999982
Precision	0.2538
Recall	0.2051
False Discovery Rate (FDR)	0.7462
False Negative Rate (FNR)	0.7949
Average Surface Distance (ASD), mm	14.889
Average Symmetric Surface Distance (ASSD), mm	14.391
Hausdorff Distance (HD), mm	41.195
95th Percentile Hausdorff Distance (HD95), mm	39.943

Table footnote:

The full-volume CBCT model was trained using 81 CBCT volumes and evaluated on a test set comprising 9 CBCT volumes. Values represent the mean performance across the test cases.

Abbreviations: DSC, Dice Similarity Coefficient; JI, Jaccard Index; FDR, False Discovery Rate; FNR, False Negative Rate; ASD, Average Surface Distance; ASSD, Average Symmetric Surface Distance; HD, Hausdorff Distance; HD95, 95th percentile Hausdorff Distance.

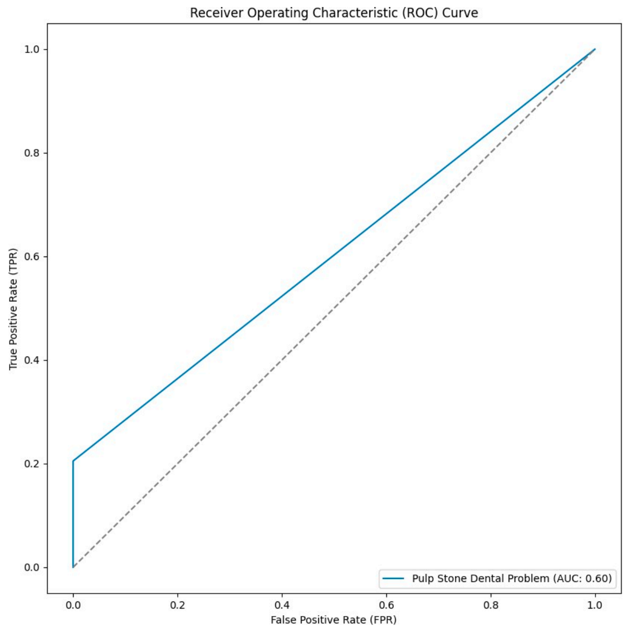

Supplementary Figure S1. Receiver operating characteristic curve of the full-volume CBCT segmentation model

Figure legend:

Receiver operating characteristic (ROC) curve of the full-volume CBCT segmentation model evaluated on nine test CBCT volumes. The area under the ROC curve (AUC) was 0.60, indicating limited voxel-level discrimination between pulp stone and background classes in uncropped full volume-CBCT volumes.

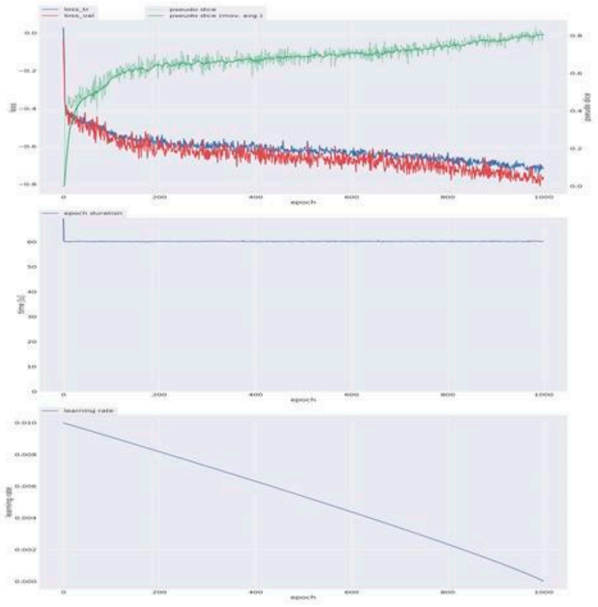

Supplementary Figure S2. Training dynamics of the full-volume CBCT nnU-Netv2 model

Figure legend:
Training dynamics of the full-volume CBCT nnU-Netv2 model over 1000 epochs. The upper panel shows the training loss, validation loss, pseudo-Dice score, and moving-average pseudo-Dice score. The middle panel presents the duration of each epoch, whereas the lower panel shows the learning-rate schedule. Although the loss values gradually decreased and the pseudo-Dice score progressively increased during training, these improvements did not translate into comparable performance on the test set, and the model exhibited limited segmentation performance.

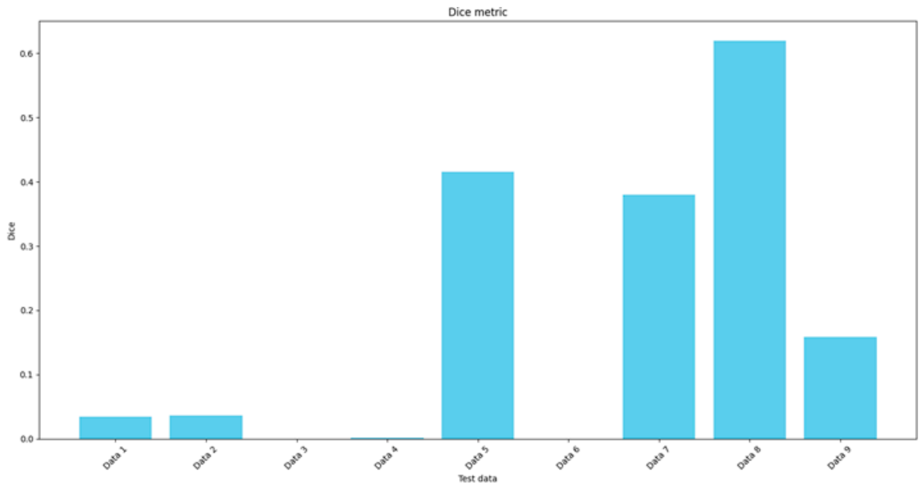

Supplementary Figure S3. Case-wise Dice Similarity Coefficient values of the full-volume CBCT model

Figure legend:

Dice Similarity Coefficient (DSC) values obtained for each of the nine test CBCT volumes evaluated using the full-volume CBCT model. Marked inter-case variability was observed, with DSC values ranging from 0.000 to 0.619. Two test cases had no voxel-level overlap between the predicted and reference segmentations and therefore yielded a DSC of 0.000.
